# Changes in Motor Competence after a Brief Physical Education Intervention Program in 4 and 5-Year-Old Preschool Children

**DOI:** 10.3390/ijerph18094988

**Published:** 2021-05-07

**Authors:** Rubén Navarro-Patón, Julien Brito-Ballester, Silvia Pueyo Villa, Vanessa Anaya, Marcos Mecías-Calvo

**Affiliations:** 1 Departamento de Didácticas Aplicadas, Facultad de Formación del Profesorado, Universidade de Santiago de Compostela, 27001 Lugo, Spain; ruben.navarro.paton@usc.es; 2 Facultad de Ciencias Sociales y Humanidades, Universidad Europea del Atlántico, 39011 Santander, Spain; julien.brito@uneatlantico.es (J.B.-B.); silvia.pueyo@uneatlantico.es (S.P.V.); vanessa.anaya@uneatlantico.es (V.A.); 3 Facultad de Ciencias de la Salud, Universidad Europea del Atlántico, 39011 Santander, Spain; 4 Centro de Investigación y Tecnología Industrial de Cantabria (CITICAN), 39011 Santander, Spain

**Keywords:** Movement Assessment Battery for Children-2 (MABC-2), childhood, specific intervention program, manual dexterity, aiming and catching, balance

## Abstract

Low motor competence (MC) can cause low participation in physical activities in preschool children, and together with a high caloric intake, it can lead to obesity. Interventions on motor skills are effective in the short term to improve MC, therefore the objectives of this study were (1) to investigate the effect of a short six-week program on levels of motor competence in preschool children, and (2) to examine the effects of gender-based intervention. A total of 156 preschool children (5.20 ± 0.54 years old) from Lugo (Spain) participated. A quasi-experimental pre–post-test design was used with a control group of 76 students. The Movement Assessment Battery for Children—2nd Edition (MABC-2) was used to collect the data. Significant differences between the control and experimental groups were found after the intervention program in aiming and catching (*p* < 0.001), balance (*p* < 0.001), the total score of eight tests (*p* < 0.001), and total percentile score (*p* < 0.001). The results regarding gender in the experimental group showed a reduction in differences with respect to the initial results except in aiming and catching, where scores were higher in boys. The data suggest that the application of specific intervention programs in MC could positively influence the improvement of MC in preschool children, thus reducing differences between genders.

## 1. Introduction

The concept of motor competence (MC) is described in scientific literature as the acquisition and improvement of skill and mastery in body movement activities [1]. The term MC has also been used to refer to the quality of each person in performing the different fundamental motor skills necessary in daily life (e.g., fastening buttons or going up or down stairs), including gross and fine motor skills [2]. Therefore, MC is a broad term that includes fundamental movement skills (FMS) ability, including locomotor, object control and stability skills [3,4]. In the case of fundamental motor skills, much of children’s learning is considered as being based on motor skills, both fine and gross [5]. Fine motor skills (e.g., writing or finger movements) are important in academic settings [6], and refer to precision movements that involve hand muscles [7]. Additionally, gross motor skills (e.g., throwing a ball or maintaining balance), require the participation of large muscle groups or even the entire body. Gross motor skills are important for children when engaging in physical activities [8]. These type of fundamental motor skills can be classified into manipulative (throwing, catching, hitting, etc.), balance (dynamic and static) and locomotive (running, sprinting, jumping, etc.) [9].

In this sense, we should keep in mind that the first years of life (up to 5 years) are an especially sensitive period for the development of physically competent children, in which motor skills must be acquired in structured learning environments, such as physical education (PE) classes or school recess [10,11], and with purpose [9]. Therefore, the acquisition of MC should be the highest priority objective for the implementation of specific programs [12], because a mature form of MC without proper practice, stimulation and feedback is less likely to be achieved [9]. For this reason, many countries have included MC as an important element in the PE curriculum in preschool education [13]. However, current World Health Organization (WHO) physical activity guidelines for preschool children (<5 years) focus on physical activity levels [14] and free play components [15], in which specific recommendations for developing MC are lacking.

On the other hand, the planning and implementation of specific interventions for the development of MC depends on the adequate identification of the child’s real level of MC [16]. This identification could be a chance of development in childhood [17]. Studies in recent years on MC in preschool children indicate that there are differences in gross skills [18,19,20,21,22,23,24,25,26] in favor of boys, and in fine skills [18,20,21,22,26,27,28,29] and balance [20,21,22,23,28] in favor of girls, and these differences between boys and girls of the same age are not uniform throughout this stage of development [28]. Therefore, early identification and intervention in children with low MC is more economically efficient and effective in reducing the problems associated with less MC development [30]. There are many assessment tools to measure MC in children [31,32], although some are product-oriented (quantitative) and the others are process-oriented (qualitative) [33]. Product-oriented tools indicate the result of skill execution (i.e., time, distance, or frequency of successful attempts) and therefore do not provide information on how the skill is performed [34,35,36]. On the other hand, process-oriented tools indicate how the skill is carried out and not so much the result; therefore, they provide specific information on which components of the task need to be improved [34,35]. The Movement Assessment Battery Test for Children—2nd Edition (MABC-2) is an assessment tool that is easy to use and interpret, safe, and feasible to apply within a school setting, which is also valid and reliable [37,38], and includes both quantitative and qualitative items [39] from the point of view of a professional (e.g., PE teachers or physical educators).

Scientific evidence indicates that children of both sexes without disabilities have low MC levels [40,41,42], therefore it is recommended that these MC interventions need to start during the preschool and early school years [33,42,43]. This must be taken into account because the FMS, which together with coordination make up the MC [44], are a set of movements under construction necessary for the performance of more complex and specific skills, for subsequent physical activities and sports [45]; they are “the equivalent of the movement to the alphabet of reading” [46]. Although it is considered that at preschool age these FMS are acquired by simple maturation, this is not the case [47], therefore, these skills must be learned, practiced and reinforced [45,48].

To improve this MC, planned interventions are needed that include duration, type of instruction [33], clear objectives, adequate practice time, and feedback [49], among others. These planned interventions are effective both in programs performed in the short term (4–8 weeks) and in the long term (≥6 months) [33,42,50,51,52,53], although shorter duration (4 weeks to 5 months) showed better results compared to longer duration (>6 months) [53] to improve the MC. In addition, the specific programs are most effective when taught by highly trained PE specialists [51], due to these interventions representing a high improvement in MC [54]. For these reasons, it is considered important to identify the type and duration of interventions so that they can help to improve MC in preschool children because there is no consensus [54]. Although some propose that they be performed at least two [52] or three times a week [55], with a minimum duration of 30 min [55], the duration of these interventions’ ranges from a minimum of 120 min [56] to a maximum of 3240 min [57]. Thus, if we want to contribute to the implementation of planned movement programs as a strategy to promote the development of MC [33,51], research on this topic should be carried out. In this sense, to the best of the authors’ knowledge, a specific intervention with all the characteristics such as those proposed in this research has not been implemented, i.e., a short-term intervention that develops and replaces regular PE classes, implemented by a specialist in PE, and that does not involve more than 40 min a week.

For all of the above, the objectives of this study were: (1) to research the effect of a short six-week program on motor competence levels in four- and five-year-old preschool children; and (2) examine the effects of the program based on gender. Thus, the hypothesis was that participation in the intervention program, as taught by a PE specialist teacher, would significantly improve the motor competence levels of all children, regardless of gender.

## 2. Materials and Methods

### 2.1. Study Design

To carry out this research a quasi-experimental design with pre- and post-test measures with a control group was created [58]. The variables of the MABC-2 were the dependent variables, comparing them according to group (control vs. experimental) and gender (boys vs. girls).

### 2.2. Participants

Four educational centers in Lugo, Galicia (Spain) were invited to participate in the study, of which two participated.

The inclusion criteria were participants who: (1) provided informed consent signed by their parents or legal guardians; (2) completed the entire process; and (3) did not suffer from illness or difficulty (physical or mental) that would prevent participation in the MABC-2 tests.

A total of 184 4–5-year-old preschool children were invited, of which 20 were excluded for not providing the informed consent signed by their parents or legal guardians, and 12 for not completing the entire process (9 preschool children were excluded because they were under the 5th percentile). Finally, the sample consisted of 152 preschool children.

### 2.3. Measurements

The Spanish version of the MABC-2 battery was used [59]. It is a valid and reliable test to identify MC changes in preschool children [39,48,59,60] with very high inter-rater reliability [61].

This battery consists of a standardized test used to identify and describe the motor function of children. For this, it is necessary to perform a series of motor tests grouped in three dimensions—manual dexterity (MD), aiming and catching (A&C) and balance (Bal)—for which the duration, depending on the age of the child and the degree of difficulty experienced, ranges between 20 and 40 min. For the three dimensions of the test and for the total score, scalar and percentile scores are provided as a function of age. The order of application of the tests must be as follows: 1st, manual skill: inserting coins; 2nd, manual skill: inserting beads; 3rd, manual skill: drawing a line; 4th, aim and catch: catching a bean bag; 5th, aiming and catching: throwing a bean bag at a target; 6th, balance (static): balancing on one leg; 7th, balance (dynamic): walking on a tiptoe; 8th, balance (dynamic): jumping on mats [39,59].

### 2.4. Procedures

The school administration was contacted, and the objective was explained. Once the schools agreed to participate in the research, the same procedure was carried out with the teachers of the different groups of preschool children. Subsequently, a study information sheet and informed consent were delivered to the parents and/or legal guardians of the school children to participate. Once accepted, the data were collected.

To correctly assess each test and to try to avoid biases, the evaluators were informed and trained following the general rules of application of the MABC-2 battery manual, recording only the quantitative data in the evaluator’s booklet, without taking into account qualitative data.

To explain each test, the evaluators always performed the same procedure: (1) description of the task; (2) demonstration by the examiner; (3) child practice following the procedure (where the examiner could correct possible errors); and (4) running the test as instructed in the manual (no instructions were given during the test). In addition, each child was individually evaluated in an isolated, bright, unobstructed, well ventilated, and noise-isolated classroom provided by the educational centers.

At the end of all tests, direct scores were obtained for each of the eight tests, and the three dimensions of the MABC-2 (i.e., MD, A&C, and Bal) and the total score (TTS) was calculated. Scalar and percentile scores (TPS) were calculated from them with the help of the manual. The scalar measures of the three dimensions, and the scalar and percentage scores of the total test score, were used in this study.

Once the students were evaluated, they were randomized by natural groups (belonging to the same group-class and school) to facilitate the program’s development.

For the experimental group, the main researcher, a PE graduate with more than 20 years of experience in educating children, and more than 10 years in training PE teachers in preschool and primary education, carried out all the intervention sessions in the indoor sports facilities of each school.

The intervention replaced PE classes in the experimental group (EG) and consisted of one 40 min session per week, for 6 weeks (i.e., 240 min). In the control group (CG), the PE teacher of each school continued with the plan without altering its programming, focusing on one of the four aspects of the PE curriculum in preschool education in Spain (i.e., the body and body image, play and movement, daily activity, and personal care and health) [13]. The exact content, duration, or frequency of the procedure followed for the control group from each school was not recorded. The teacher was unaware of the intervention that was carried out with the experimental group and did not help in its application either.

Each session of the EG began with a warm-up or welcome activity (5 min), three or four tasks related to the skill to be developed (manual dexterity, pointing and chatting or balance; 30 min) and a cool-down or goodbye activity (5 min). The sessions were structured based on the objectives as follows (Table 1): Session 1: Introduce manual dexterity, balance and the overall skills of throwing and catching games; Session 2: Improve fine motor and manual dexterity, jot down tasks, grip and balance; Session 3: Develop manual dexterity with both hands and practice the tasks of catching and receiving various objects; Session 4: Improve fine motor skills in both hands. Develop aim and precision when throwing objects; Session 5: Work on manual dexterity and fine motor skills, develop static and dynamic balance; Session 6: Remember through the motor circuit, tasks and games performed in previous sessions. Work with manual dexterity, aiming, grip and balancing, following the same distribution proposed by Navarro et al. [62].

The day after the end of the intervention, MC was re-evaluated with the MABC-2 battery in both CG and EG.

### 2.5. Ethics

The research was approved by the Ethics Committee of the national EDUCA (code 22019) platform, according to the standards established in the Declaration of Helsinki.

### 2.6. Statistical Analysis

IBM SPSS version 25 software (SPSS v.25, IBM Corporation, New York, NY, USA) was used for statistical analysis, and the level of significance was set at *p* < 0.05. First, the data were found to follow a normal distribution using the Kolmogorov–Smirnov test. The independent samples *t*-test was used to assess the differences of the Control (CG) and Experimental (EG) groups in the MABC-2 battery tests (i.e., manual dexterity, aiming and catching, balance, total test score and total percentile score), before the intervention program to establish that the groups were equivalent. In addition, a chi-squared analysis was performed to compare the distribution of the participants according to gender. Once the intervention process was applied in the PE classes, the *t*-test of related samples was used to evaluate the changes produced in each group (CG vs. EG) and an independent samples *t*-test to investigate the difference in the pre–post change between each group (CG and EG) was used. Statistical power was expressed by the Cohen´s d statistic, with d = 0.20 small, d = 0.50 moderate and d = 0.80 large.

## 3. Results

A total of 152 healthy preschool children were evaluated, of whom 70 (46.10%) were girls and 82 (53.9%) were boys aged 4–5 years old (mean = 5.20; SD = 0.54). The distribution of the participants was 76 preschool children from CG, and 76 preschool children from EG.

The normality test revealed that the data followed a normal distribution, i.e., manual dexterity (*p* = 0.115), aiming and catching (*p* = 0.392), balance (*p* = 0.223), total eight test score (*p* = 0.107), and total percentile score (*p* = 0.060).

### 3.1. Baseline Characteristics

The baseline characteristics of the MABC-2 are outlined in Table 2. Participants in CG and EG were similar at baseline for manual dexterity (*p* = 0.905), aiming and catching (*p* = 0.055), balance (*p* = 0.656), total eight test score (*p* = 0.196), and total percentile score (*p* = 0.190). Furthermore, the distribution of participants by gender was similar in both groups (*p* > 0.05).

The overall results of the previous test (before the intervention) for the total sample, according to gender, were (Table 2): manual dexterity (*p* < 0.001), aiming and catching (*p* > 0.050), balance (*p* < 0.001), total eight test score (*p* < 0.001) and total percentile score (*p* < 0.001), with all test scores higher for girls than boys, except for aiming and catching. In the CG, the results were: manual dexterity (*p* = 0.443), aiming and catching (*p* = 0.210), balance (*p* = 0.013), total eight test score (*p* = 0.296) and total percentile score (*p* = 0.309), with all test scores higher for girls than boys. Lastly, in EG, the results were: manual dexterity (*p* < 0.001), aiming and catching (*p* = 0.993), balance (*p* = 0.001), total eight test score (*p* < 0.001) and total percentile score (*p* < 0.001), with the scores of all the tests higher in girls than in boys, as in the CG.

### 3.2. Control Group Outcomes

**Figure 1:**
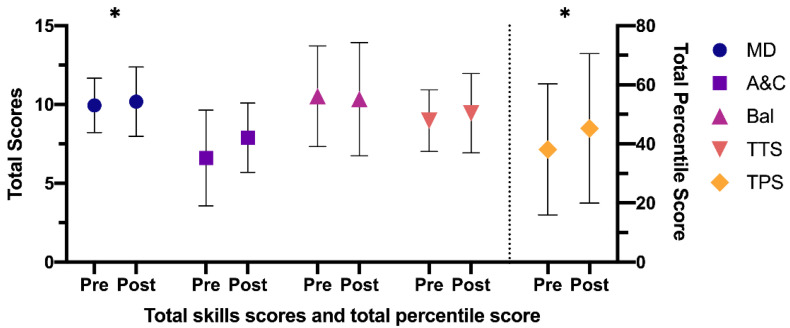
Differences between pre- and post-tests in the CG. CG: Control Group; MD: manual dexterity; A&C: aiming and catching; Bal: balance; TTS: total test score; TPS: total percentile score. Note: * *p* < 0.001 difference between pre- and post-test.

After the intervention program, the results obtained in the CG were: manual dexterity—mean difference: −0.24 (95% CI: −0.73–0.26), t (75) = −0.945; *p* = 0.347, *d* = 0.11—aiming and catching—mean difference: −1.28 (95% CI: −1.92–0.65), t (75) = −4.058; *p* < 0.001, *d* = 0.46—balance—mean difference: 0.18 (95% CI: −0.49–0.86), t (75) = 0.542; *p* = 0,590, *d* = 0.06—total eight test score—mean difference: −0.47 (95% CI: −1.02 –0.07), t (75) = −1.715; *p* = 0.091, *d* = 0.19—and total percentile score—mean difference: −7.15 (95% CI: −12.90–1.41), t (75) = −2.479; *p* < 0.001, *d* = 0.28. All test scores were higher in the post-test compared to the pre-test, except in balance, which were lower, although not significantly (Figure 1).

**Figure 2:**
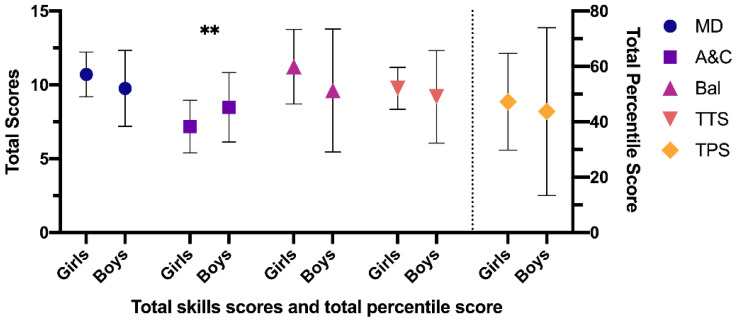
Differences between girls and boys post-test in the CG. CG: control group; MD: manual dexterity; A&C: aiming and catching; Bal: balance; TTS: total test score; TPS: total percentile score. Note: ** *p* < 0.05 difference between girls and boys.

After the intervention program, the results obtained in the CG regarding gender were: manual dexterity (*p* = 0.063), aiming and catching (*p* = 0.010), balance (*p* = 0.051), total eight test score (*p* = 0.326) and total percentile score (*p* = 0.544), reducing the differences with respect to the initial results in all dimensions except aiming and catching, which increased the differences between boys and girls due to increased scores in boys (8.47; SD = 2.35) compared to girls (7.17; SD 1.78) (Figure 2).

### 3.3. Experimental Group Outcomes

**Figure 3:**
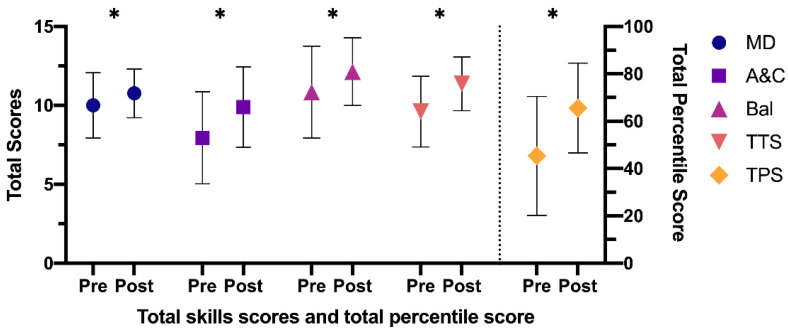
Differences between pre- and post-test in the EG. EG: experimental group; MD: manual dexterity; A&C: aiming and catching; Bal: balance; TTS: total test score; TPS: total percentile score. Note: * *p* < 0.001 different between pre- and post-test.

The results obtained regarding the difference between the pre- and post-test, in the EG, at the overall level (Figure 3) were: manual dexterity—mean difference: −0.76 (95% CI: −1.16 to −0.37), t (75) = −3.869; *p* < 0.001, *d* = 0.45—aiming and catching—mean difference: −1.95 (95% CI: −2.48 to −1.41), t (75) = −7.281; *p* < 0.001, *d* = 0.84—balance—mean difference: −1.29 (95% CI: −1.86 to −0.71), t (75) = −4.427; *p* < 0.001, *d* = 0.51—total eight test score—mean difference: −1.76 (95% CI: −2.16 to −1.37), t (75) = −8.939; *p* < 0.001, *d* = 1.03—and total percentile score—mean difference: −20.21 (95% CI: −24.69 to −15.73), t (75) = −8.983; *p* < 0,001, *d* = 1.04. In this case, the scores increased significantly after the application of a specific intervention program in the PE classes.

**Figure 4:**
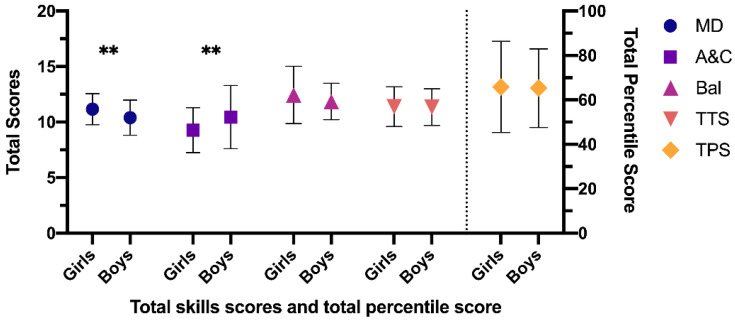
Differences between girls vs. boys post-test in the EG. EG: experimental group; MD: manual dexterity; A&C: aiming and catching; Bal: balance; TTS: total test score; TPS: total percentile score. Note: ** *p* < 0.05 difference between girls and boys.

After the intervention program, the results obtained in the EG regarding gender were: manual dexterity (*p* = 0.029), aiming and catching (*p* = 0.045), balance (*p* = 0.230), total eight test score (*p* = 0.922) and total percentile score (*p* = 0.903), reducing the differences with respect to the initial results in all dimensions, except in aiming and catching, where the differences between boys and girls increased due to increased scores in boys (mean 10.45; SD = 2.85) with respect to girls (mean 9.27; SD = 2.02) (Figure 4).

### 3.4. Experimental Group vs. Control Group Outcomes

**Figure 5:**
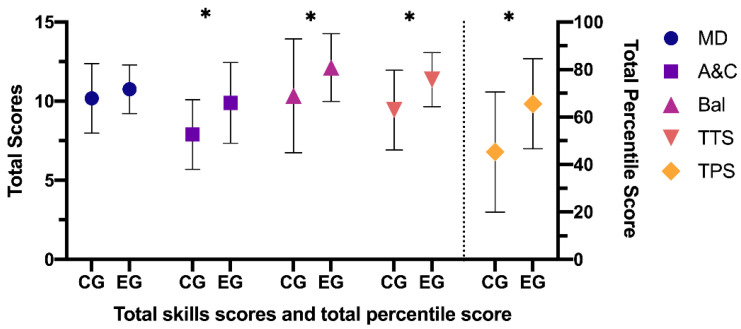
Differences between CG and EG after the application of the training program. CG: control group; EG: experimental group; MD: manual dexterity; A&C: aiming and catching; Bal: balance; TTS: total test score; TPS: total percentile score. Note: * *p* < 0.001 difference between CG and EG.

The results of the comparisons between the CG and the EG after the application of the training program (Figure 5) were: manual dexterity—CG: (M = 10.18, SD = 2.20) vs. EG (M = 10.76, SD = 1.53), (95% CI: −0.03–1.18), t (150) = 1.879; *p* = 0.062, *d* = 0.30—aiming and catching—CG: (M = 7.89, SD = 2.20) vs. EG (M = 9.89, SD = 2.54), (95% CI: 1.24–2.76), t (150) = 5.178; *p* < 0.001, *d* = 0.84—balance—CG: (M = 10.34, SD = 3.59) vs. EG (M = 12.13, SD = 2.14), (95% CI: 0.84–2.74), t (150) = 3.727; *p* < 0.001, *d* = 0.61—total eight test score—CG: (M = 9.44, SD = 2.51) vs. EG (M = 11.36, SD = 1.79), (95% CI: 1.23–2.61), t (150) = 5.512; *p* < 0.001, *d* = 0.88—total percentile score—CG: (M = 45.26, SD = 25.29) vs. EG (M = 65.55, SE = 18.95), (95% CI: 13.12–27.45), t (150) = 5.597; *p* < 0.001, *d* = 0.91.

## 4. Discussion

To the best of the authors’ knowledge, this is the first study to examine whether a short-term intervention program in MC can provide immediate improvements in the levels of manual dexterity, aiming and catching, balance, total test score, and total percentile score of Galician preschool children, and if its effects are different according to gender. The results of this study suggest that children are more likely to have better MC if they receive specialized and specific instruction in PE classes from a specialist [40,51,53,55] than if it is taught by a preschool education teacher through general activities in PE classes or free play [63].

Our results show that for an improvement in MC, a specific intervention is necessary through a program of planned and adequate motor activities to teach and practice gross (i.e., locomotor and object control) and fine (i.e., dexterity manual) motor skills and balance [33,55,64].

Before the intervention, both CG and EG presented similar MC without statistical differences in each of the overall studied skills. There were differences between boys and girls in both groups according to gender. The girls’ scores were higher in MD, Bal, TTS and TPS overall, and in each of the groups (CG and EG), as in previous studies [22,26,27,28,29,65,66]. Scores in A&C were similar between boys and girls, results that do not agree with the findings to date, because boys tend to display higher levels of mobile control and manipulation skills [25,46,48,66,67,68,69].

Once the intervention period had ended, the scores in the different dimensions in the CG increased, although not as significantly as in the EG. In contrast to the motor intervention, the benefits were only found in aiming and catching and, consequently, in the total percentile score. These advances in MC may have occurred as part of the normal growth and development and/or maturation of preschool-aged children [70]. Therefore, as individuals mature, their MC can be modified without practice, although with few significant improvements [54]. The results found in the CG surprised us because it was expected that the preschool children would improve their MC, because both the PE classes and the motor intervention classes included opportunities for structured movement and we expected similar results between both groups [54]. These results in our study could be partially explained by the fact that the teacher in charge of PE classes was not a specialist in this area and therefore would not have knowledge about the design and implementation of specific movement activities [33,53]. In this group, the differences between boys and girls were maintained in all dimensions except A&C, in which these differences became significant as boys obtained better scores [25,46,48,66,67,68,69]. The differences by gender continued to be maintained, although the scores improved in boys and girls compared to the initial assessment. These results agree with those found by Bolger et al. [71] and Cohen et al. [72], who indicated that after eight months of PE classes, the differences in MC had not increased significantly. This may be due to country factors, such as the one in which this study was conducted, where PE in preschool children is taught by generalist teachers, most of whom have limited specific training in PE [33,53,73], as in our case.

In the EG, once the specific program was applied by a specialist PE teacher, the scores of the different dimensions increased significantly, by which we can say that the applied intervention program produces improvements in MC [33,51,53,55,74,75,76,77], demonstrating that motor interventions are more effective than PE classes [54], consolidating the position that the participation of experts in this area is needed to design and implement PE classes to improve MC.

If we analyze the pre–post-test differences according to gender, contrary to what happened in the CG, the differences before the intervention decreased (MD), disappeared (Bal, TTS and TPS), or even appeared in favor of boys (A&C) [70,78,79], because a structured program on MC can benefit children in these skills [51,74,80]. These results coincide, in part, with the results found by Jimenez-Diaz et al. [54] that indicate that improvements in MC occur after a specific intervention in PE, regardless of the gender of the participants and the duration of the program. Thus, specific motor interventions have the characteristic of being adequate, from the point of view of development and implementation, for the age of the participants [33], having a positive effect on all evaluated components of MC [40,51,53,54,55].

When comparing the CG and EG after the intervention program was applied, statistically significant differences appeared [75], and large and medium effect sizes can be reported for the changes in MABC-2 scores [12,33,55,64]. Even though this intervention was for a weekly session and for only six weeks, not following the parameters of other interventions carried out [53,55,56,57], there were improvements in EG that could be explained by the ceiling effect [51], which indicates that preschool children could achieve better performance in the early stages of the intervention in such a way that a longer intervention time does not translate into better performance [54]. Therefore, brief interventions, such as ours, can produce improvements in the MC of preschool children in such a way that the total duration (in minutes) and the frequency are moderators in improvement of the MC [33].

Differences occurred in A&C, and Bal, and consequently, as these differences increased, so did the total test scores and the total percentile scores. These results agree with other reports from previous literature [51,74,75,76,80], but not with the results of Kelly et al. [81]. The only variable in which these differences were not significant was in MD, which still scored higher in the EG than in the CG. These results could be due to the fact that the quality and specificity of the movement programs carried out by a PE specialist could be better than the PE classes led by non-specialists in the area [53].

## 5. Conclusions

The findings support the hypothesis that participating in the six-week intervention program would significantly improve the motor competence levels of all children, regardless of gender. However, whether these improvements can be sustained over time should be researched. The results of this study suggest that a specific intervention on motor competence in short-term preschoolers, carried out by PE specialists, can significantly improve manual dexterity, aiming and catching, balance, total test scores, and total percentile scores on the MABC-2 post-intervention tests.

## 6. Study Limitations and Practical Applications

In addition to the contributions of this study, certain limitations must be taken into account that should cause the results to be viewed with caution. No follow-up was performed to determine the effectiveness of this long-term intervention program. Additionally, the sample size may be relatively small compared to other studies. On the other hand, the multiple personal and environmental factors [82], which can affect MC at any given time, were not taken into account. Furthermore, there is currently no quality assessment tool to specifically evaluate MC programs, therefore we do not know if this intervention would have the same effect elsewhere.

From an educational point of view, although the studies carried out to date recommend that an intervention in MC achieves better results when it is taught by PE specialists, non-specialist teachers should propose specific programs for the development of MC (PE classes, recess or breaks in the classroom) because this could contribute to the practice of physical activity in preschoolers, by which they should be trained in this specialty. PE classes should promote participation by all students, and thus allow a perceived success and competence, which will lead to increased practice.

Given that the girls after the intervention continued to show worse mastery on the ability to control objects than the boys, studies should be carried out in which a gender approach is proposed in these interventions.

## Figures and Tables

**Table 1:** Objectives and tasks performed in each of the 6 sessions.

Session Number	Objectives	Tasks (Skills)
Session 1 “I explore my body”	Introduce manual dexterity, balance and global throwing and catching skills through games	“We play with the tweezers” (manual dexterity) “Balance chase game” (balance) “Do not fall!” (aiming and catching) “Manual golf” (aiming and catching) “The jumping kangaroos” (balance)
Session 2 “I develop my motor skills”	Improve fine motor and manual dexterity, jot down tasks, grasp and balance	“Wrap the giraffe” (manual dexterity) “Shooting into the tunnel” (aiming and catching) “Balance circuit” (balance)
Session 3 “The art of catching”	Develop manual dexterity with both hands and practice the tasks of catching and receiving various objects	“Chinese carriers” (manual dexterity) “Catch practice” (catching) “Catch and win” (catching) “Molded animals” (manual dexterity)
Session 4 “Sharpen your aim”	Improve fine motor skills in both hands. Develop aim and precision when throwing objects	“The coin catcher” (manual dexterity) “Aim for the bullseye” (aiming) “Double throw” (aiming and catching) “The labyrinth” (manual dexterity)
Session 5: “Circus tightrope walkers”	Work on manual dexterity and fine motor skills, develop static and dynamic balance.	“Paste-stickers” (manual dexterity) “The stilts” (balance) “The rescue” (balance and aiming and catching) “The endless line” (balance) “To pick up!”
Session 6: “Motor circuits”	Remember through the motor circuit, tasks and games performed in previous sessions. Work with manual dexterity, aiming, grip and balancing	“The circuit” (manual dexterity; aiming and catching; balance) “Circuit 1” (manual dexterity; aiming and catching; balance) “Circuit 2” (manual dexterity; aiming and catching; balance)

**Table 2:** MABC-2 baseline characteristics of study participants.

Total Scores	Total (*n* = 152)			Control Group (*n* = 76)			Experimental Group (*n* = 76)		
	All	Male (*n* = 82)	Female (*n* = 70)	All	Male (*n* = 42)	Female (*n* = 34)	All	Male (*n* = 40)	Female (*n* = 36)
Manual Dexterity	9.97 ± 1.89	9.46 ± 1.84	10.57 ± 1.79	9.94 ± 1.72	9.80 ± 1.51	10.11 ± 1.96	10.00 ± 2.06	9.10 ± 2.09	11.00 ± 1.51
Total score for aiming and catching	7.27 ± 3.04	7.46 ± 2.94	7.05 ± 3.15	6.60 ± 3.03	7.00 ± 2.99	6.11 ± 3.05	7.94 ± 2.92	7.95 ± 2.85	7.94 ± 3.03
Total score for balance	10.68 ± 3.05	9.78 ± 3.00	11.74 ± 2.76	10.52 ± 3.18	9.71 ± 3.12	11.52 ± 3.02	10.84 ± 2.91	9.85 ± 2.90	11.94 ± 2.54
Total 8 test Score	9.28 ± 2.11	8.73± 1.85	9.94 ± 2.21	8.97 ± 1.95	8.76 ± 1.87	9.23 ± 2.04	9.60 ± 2.23	8.70 ± 1.87	10.61 ± 2.19
Total Percentile Score	41.72 ± 23.87	35.48 ± 21.01	49.02 ± 25.07	38.10 ± 22.16	35.76 ± 21.51	41.00 ± 22.94	45.34 ± 25.09	35.20 ± 20.73	56.61 ± 24.93

Data are presented as the mean ± standard deviation of the mean.

## Data Availability

The data presented in this study are not available, in accordance with Regulation (EU) of the European Parliament and of the Council 2016/679 of 27 April 2016 regarding the protection of natural persons with regard to the processing of personal data and the free circulation of these data (RGPD).
